# Digestibility of Protein and Iron Availability from Enriched Legume Sprouts

**DOI:** 10.1007/s11130-023-01045-x

**Published:** 2023-02-02

**Authors:** Magdalena Zielińska-Dawidziak, Wojciech Białas, Dorota Piasecka-Kwiatkowska, Halina Staniek, Przemysław Niedzielski

**Affiliations:** 1 Department of Food Biochemistry and Analysis, Faculty of Food Science and Nutrition, Poznań University of Life Sciences, Poznań, Poland; 2 Department of Biotechnology and Food Microbiology, Faculty of Food Science and Nutrition, Poznań University of Life Sciences, Poznań, Poland; 3 Department of Human Nutrition and Dietetics, Faculty of Food Science and Nutrition, Poznań University of Life Sciences, Poznań, Poland; 4 Department of Analytical Chemistry, Faculty of Chemistry, Adam Mickiewicz University, Poznan, Poland

**Keywords:** Ferritin, Digestibility, Soybean, Lupine, Fortified sprouts

## Abstract

Plant ferritin is suggested as a good source of iron for human. Usually present in trace amounts, it was induced in legumes seeds by their sprouting in FeSO_4_ solution. Fortified sprouts were digested in the *in vitro* model of the human gastrointestinal tract. ~49% of lupine and ~ 45% of soy proteins were extracted into gastric fluid and next ~ 12% and only ~ 1% into intestine fluid from lupine and soybean, respectively. Gastric digestion released mainly ferrous iron (~ 85% from lupine and ~ 95% in soybean sprouts). Complexed iron constituted ~ 43% of total iron in intestine after lupine digestion and ~ 55% after soybean digestion. Intestine digestion doubled the total iron released from lupine sprouts (from ~ 21% up to 38%), while in soybean it increased from ~ 16% up to ~ 23%. Ferritin presence was confirmed by the specific antibodies in digestive fluids, but it is only partially extracted from sprouts during *in vitro* digestion.

**Supplementary Information:**

The online version contains supplementary material available at 10.1007/s11130-023-01045-x.

## Introduction

Iron deficiency anemia (IDA) is the most common type of anemia (about 50% incidence) affecting more than 1.2 billion in 2016, particularly infants, children, adolescents, pregnant women, the elderly or people suffering from eating disorders [1]. To treat IDA, diet modification and supplements intake are recommended. However, iron supplementation (tablets application as well as enriched food consumption) may bring numerous side effects, such as constipation, diarrhea, abdominal pain and nausea [2]. An alternative to this type of strategy may be the use of food biofortified in iron, such as legumes sprouts enriched in ferritin [3, 4]. Ferritin is a protein able to contain up to 4,500 Fe ions, and it also protects iron from chelating agents present in the diet. However, studies on the bioavailability of ferritin iron are contradictory [5–8]. *In vivo * results suggest higher stability of ferritin than *in vitro* studies [5, 8, 9]. This may be due to the fact that pure, isolated ferritin is usually used in *in vitro* studies, whereas food rich in ferritin is often part of the human diet in *in vivo* studies. Clinical studies are still considered a “gold standard” of nutritional research [10]. However, *in vitro* methods are characterized by a significantly shorter time, the ease of sampling a large number of samples, lower costs and labor consumption, a guarantee of repeatability and no ethical restrictions. That is why still *in vitro* methods simulating digestive processes are widely used in research on the behavior of food and pharmaceutical products in the digestive tract [10].

The aim of the presented research was to study the availability of iron and proteins from the sprouts enriched in iron in a simplified *in vitro* digestion model. It is hypothesized that food constituents may have a protective effect on ferritin during digestion and increase its potential as a source of bioavailable iron.

## Materials and Methods

### Sprouts Preparation

Soy (*Glycine max*, Augusta variety) and lupine seeds (*Lupinus luteus*, Lord var.) from the Poznan University of Life Sciences cultivations were used for the preparation of iron-enriched sprouts.

The sprouting process was carried out according to the procedure presented previously [3, 11]. The seeds were cultured for seven days under controlled conditions and watered with 25 mM FeSO_4_ (POCh, Poland) from the third day of cultivation. Then the sprouts were dried, until their moisture was decreased to below 14%, and milled. Two manners of material drying were applied: continuous drying in 35^o^C, or two-stage drying: 5 h in 80^o^C, followed by drying in 35^o^C. As a control sample the sprouts not fortified in iron (watered only with distilled water with addition of 0.7 mM Ca^2+^ and 0.4 mM Mg^2+^), prepared in the same conditions, were applied. The sprouts were cultivated and dried in 7-fold repetition, mixed, and stored in an airtight glass container prior to further analysis.

### *In Vitro* Digestion Experiment

The digestion of the prepared sprouts was done in six repetitions by an *in vitro* method, simulating two-stage multi-enzymatic (gastric and intestinal) digestion [12, 13]. The digestion in the oral cavity was not considered, because it has no effect on their digestibility (the studied material is rich in protein, poor in starch). The large intestine stage was also omitted, as not important at this stage of iron availability studies.

A studied sample (0.5 g) was introduced into distilled water (50 mL) containing pepsin (60,000 U) (Sigma) and pH of the mixture was lowered to 2.0 with 1 M HCl. Gastric digestion was carried out for 2 h, at 37^o^C. Then, the pH of the solution was adjusted to 7.4 and a solution containing pancreatic-intestine extract (0.005 g, Sigma) and bile salts (0.03 g, Sigma) in 5 ml 0.1 M NaHCO_3_ (POCh, Poland) was added. The digestion was again performed at 37 °C for 2 h (pancreatic extract concentration was based on the manufacturer’s data). The solution was centrifuged and the remaining extracted and not digested proteins were precipitated with trichloroacetic acid (TCA) (Sigma, Poland). In the sample prepared in this way, protein nitrogen was determined using the Kjeldahl [14] method and was related in percentage to the amount of protein nitrogen introduced with the sample into the digestion test. Additionally, the concentration of protein released from the tested material into digestive fluids at individual stages of digestion (*i.e*., stomach and intestine) before precipitation by TCA was determined with the Bradford method [15].

### Iron Determination

The iron released during digestion from the sprouted seeds was determined as total iron and iron in ionic form. The total iron determination was conducted with atomic absorption spectrometry with air-acetylene flame atomization, while the ionic iron was determined as Fe(II) and Fe(III) forms by the colorimetric procedure. Fe(II) ions content was determined in reaction with 2,2′-bipyridyl (Sigma, Poland) in the environment of acetate buffer (pH 4.5) (Sigma, Poland), while Fe(III) in reaction with thiocyanate (Supelco, Poland) in the environment of hydrochloric acid (Sigma, Poland) (pH < 2) using photometry (wavelength 470 nm). For each sample, a blank sample was prepared and the obtained value was used to correct the interference of the sample color [16].

The complexed iron content (in the studied preparation considered as iron bound mainly in ferritin form) was calculated from the difference between the total iron content and sum of ionic iron (Fe(II) and Fe(III) contents).

### Lupine Ferritin Standard Preparation

Purification of lupine ferritin was made using Korcz and Twardowski method [17]. The procedure included two-step salting out of protein from the homogenized sprouts (in the buffer containing 50 mM Tris-HCI, pH 8,0, and 30 mM NaCl). Next, the ferritin was purified by fourfold ultracentrifugation (100,000x *g*/120 min).

### FPLC Separation

The chromatographic studies were performed with an AKTA Explorer 100 Air System (Amersham Pharmacia Biotech, Uppsala, Sweden). A HiLoad 26/60 Superdex 200 column from Amersham Pharmacia Biotech (Uppsala, Sweden) was used. Assays on digested samples were performed at room temperature, at a flow rate of 2 ml/min. A 15 ml sample was eluted with 0.05 M of phosphate buffer pH 7 containing NaCl in a concentration of 0.5 M. The chromatographic mobile phase prior to use, and all samples before injection into the column, were filtered through a membrane filter (0.45 μm, Millipore). During the chromatographic run, fractions were collected in volumes of 12 ml. Absorbance at 280 nm was applied for protein detection.

The column was calibrated with the selected standard proteins included in the low molecular weight (LMW) and high molecular weight (HMW) range calibration kits (Cytivia, USA). The following proteins were used: Aprotinin (6500 Da), Carbonic anhydrase (29 000 Da), Ovalbumin (43 000 Da), Conalbumin (75000 Da), Aldolase (158000 Da), Ferritin from Horse spleen (440 000 Da) and Blue Dextran (2000000 Da). A calibration curve was prepared by measuring the elution volumes (Ve) of standards, calculating their corresponding partition coefficient (Kav values), and plotting their Kav values vs. the logarithm of their molecular weight. The Kav was calculated with the following Eq. (1):

1 $${\text{K}}_{av}=\frac{{\text{V}}_{\text{e}}- {\text{V}}_{0}}{{\text{V}}_{\text{t}}- {\text{V}}_{0}}$$

where: V_e_ is elution volume for the standard (mL), V_0_ is column void volume = elution volume for Blue Dextran 2000 (mL), Vt is total column volume (mL). Calibration data is included in Supplementary Materials.

The fractions collected after separation by size exclusion chromatography with a retention volume similar to the lupine ferritin standard were further analyzed (SDS-PAGE, western blot and slot blot).

### SDS-Page Separation

The fraction separated by FPLC method was subjected to electrophoresis in 14% polyacrylamide gels under denaturing conditions [18]. Samples obtained from soy chromatography were directly denatured, while lupine samples were first concentrated 10 times through filters with point cut-off 3 kDa (Amicon Ultra, Millipore Ltd.). Gels were dyed with Coomassie Brilliant Blue and documented using CLIQS (TotalLabQuant, GB). Molecular mass of the detected protein was determined by reference to molecular mass marker in range 20–120 kDa (Thermo Fisher Scientific, Waltham, USA).

## Ferritin Immunodetection

### Slot-blot Analysis

The collected fractions after FPLC analysis were applied on the PVDF membrane (Immobilon-P 0,45 μm, Merck Millipore Ltd., Poland). The applied volume was 200 *µ*l for lupine fractions and 20 *µ*l for soy fractions. 1% BSA in Tris-buffered saline, pH 7.4 was used as the blocking agent (1-h incubation). Next, the membrane was incubated for 2 h with the goat sera containing anti-lupine ferritin antibodies, diluted 1:100 (provided for the research by the Institute of Bioorganic Chemistry of the Polish Academy of Sciences, Poznań). As a secondary antibody, rabbit anti-goat IgG polyclonal antibody, marked with horseradish peroxidase was applied for 2 h in 1:1000 dilution (Invitrogen, USA). Detection of protein-bound antibodies was performed with diaminobenzidine (Sigma-Aldrich, USA) in 20 min.

### Western Blot Analysis

Protein fractions separated by SDS-PAGE electrophoresis were also transferred by a semi-dry electrotransfer (200 mA current for 30 min. and 120 mA for 90 min.) to a polyvinylidene difluoride membrane (Immobilon-P 0,45 μm, Merck Millipore Ltd., Poland). The same antibodies and method of detection were used as presented above for the slot-blot analysis. The membranes were analysed using the CLIQS program (TotalLab Quant, UK).

### Statistical Analysis

The necessary statistical analyses were performed using Statistica 13.0 (StatSoft, USA). All numerical data were presented as mean ± SD. The statistical significance of the difference between the control and the treated sample was assessed by one-way ANOVA and post-hoc Tukey’s tests. Results were considered statistically significant at *P *< 0.05.

## Results and Discussion

### Protein Digestibility

The protein content in the tested lupine seeds (43.61 ± 0.22 g/100 g d.m. (dry matter)) and sprouts (47.76 ± 0.40 g/100 g d.m.) is over 4% higher compared to soybean seeds (39.18 ± 0.19 g/100 g d.m.) and sprouts (42.51 ± 0.13 g/100 g d.m.). The protein content may vary depending on the seed variety and conditions of seed cultivation [3]. Only the S.Fe.35 (soybean sprouts enriched in iron, dried continuously in 35^o^C) variant differed slightly, but statistically significantly (Table 1).

**Table 1:** Total protein and soluble protein balance in the test material before and after *in vitro* digestion (g/d.m.) of soy (S) and lupine (L) sprouts; 0 – control sample, Fe – samples enriched in iron; 35℃ – sprouts dried in the temperature 35℃, 80℃ – sprouts dried in the temperature 80℃

Sample	Content of total protein in the weight of material introduced into digestion [g/100 g d.m.]	Content of total protein in sample [g/100 g d.m.]				Total digestibility [%]	Soluble protein content determined in fluids after digestion by Bradford method [µg/ml]	
		after the 1st stage of digestion		after the 2nd stage of digestion				
		Fluid	Sediment	Fluid	Sediment		after the 1st stage	after the 2nd stage
L.0.35	2.63 ± 0.01 ^a *^	1.28 ± 0.00 ^a^	1.35 ± 0.02 ^b^	1.40 ± 0.03 ^a^	1.22 ± 0.03 ^d^	53.23 ^a^	364.17 ± 5.20 ^b^	298.33 ± 1.44 ^a^
L.0.80	2.61 ± 0.01 ^a^	1.26 ± 0.01 ^a^	1.35 ± 0.02 ^b^	1.65 ± 0.01 ^c^	0.96 ± 0.02 ^b^	63.22 ^b^	346.67 ± 1.44 ^a^	333.33 ± 3.82 ^b^
L.Fe.35	2.65 ± 0.00 ^a^	1.25 ± 0.01 ^a^	1.40 ± 0.01 ^b^	1.84 ± 0.02 ^d^	0.81 ± 0.02 ^a^	69.4 ^c^	350.83 ± 5.00 ^a^	465.00 ± 11.55 ^d^
L.Fe.80	2.63 ± 0.02 ^a^	1.37 ± 0.02 ^b^	1.26 ± 0.03 ^a^	1.51 ± 0.02 ^b^	1.12 ± 0.04 ^c^	57.4 ^d^	375.00 ± 2.89 ^c^	379.17 ± 11.46 ^c^
S.0.35	2.37 ± 0.01 ^a^	1.16 ± 0.01 ^c^	1.21 ± 0.01 ^a^	1.06 ± 0.01 ^a^	1.31 ± 0.01 ^b^	44.73 ^a^	388.33 ± 13.23 ^c^	462.50 ± 10.10 ^c^
S.0.80	2.37 ± 0.01 ^a^	1.07 ± 0.01 ^b^	1.30 ± 0.01 ^b^	1.11 ± 0.01 ^b^	1.26 ± 0.01 ^a^	46.84 ^b^	305.00 ± 6.29 ^a^	370.83 ± 6.29 ^a^
S.Fe.35	2.39 ± 0.01 ^b^	0.91 ± 0.01 ^a^	1.48 ± 0.01 ^c^	1.14 ± 0.01 ^b^	1.25 ± 0.01 ^a^	47.70 ^b^	320.83 ± 4.33 ^b^	550.00 ± 7.64 ^d^
S.Fe.80	2.36 ± 0.01 ^a^	1.17 ± 0.01 ^c^	1.19 ± 0.01 ^a^	1.11 ± 0.01 ^b^	1.25 ± 0.01 ^a^	47.04 ^b^	411.67 ± 7.64 ^d^	425.00 ± 14.65 ^b^

* a, b, c, d – statistically determined (with the Tukey Test) homogeneous groups (separately for results of soybean and lupine in the column)

The availability of nutrients for the human body depends not only on their content in the digested material, but also on the extractivity of the components from digested material at individual stages of digestion. Ingredients remaining in undigested material are excreted with feces. Absorption is possible for those substances which, after being extracted in the stomach or intestine, are dissolved in digestive fluids. Thus, the nitrogen compounds (recalculated into total protein) released from the digested material were studied.

The most of total protein during digestion was released into the gastric fluid from the L.Fe.80 (lupine sprouts enriched in iron and dried in 80ºC for the first 5 h) and S.Fe.80 (enriched soy sprouts dried in 80ºC for the first 5 h) variant, and the least from L.Fe.35 and S.Fe.35 (lupine and soy sprouts enriched in iron and dried continuously in 35ºC). It may be explained by the denaturation of proteins during drying at 80ºC, which facilitates their hydrolysis, but also extraction in the stomach.

On the other hand, as a result of total digestion, the most total protein is secreted into intestinal fluid during digestion of L.Fe.35 and S.Fe.35, while the least is in L.0.35 and S.0.35 (not enriched soy and lupine sprouts dried in 35ºC). Most of protein is extracted to the gastric fluid (respectively ~ 49% for lupine and ~ 45% for soybean sprouts) and average increase in the total content of protein in the intestinal fluid (*i.e*., total digestibility) is up to ~ 61% for lupine and only up to ~ 46% for soybean sprouts. These results suggest that proteins of lupine sprouts were more digestible, and it may result from the decreased content of trypsin inhibitors compared to soy. Lupine is usually indicated as a legume with trace content of trypsin inhibitor activity [13]. However, both the thermal treatment and the sprouting processes reduce the activity of these inhibitors in the material [19, 20].

Total protein content informs us about extractivity of nitrogen compounds from the digested material. Intestinal enterocytes absorb mainly free amino acids or very short peptides. Only few protein are absorbed via endocytosis, e.g. ferritin [7, 8]. Thus, in order to distinguish the amount of proteins that are released during digestion from amino acids, short peptides and nucleotides, determination of the protein extracted from the tested material was done by the Bradford method. The method allows to determine peptides/proteins that exceed 3–5 kDa [21], i.e. peptides composed of at least ~ 27 amino acids. The highest increase in the soluble protein content in intestine fluid compared to stomach fluid was observed in samples fortified in iron and dried in 35^o^C (L.Fe.35 - ~35%, S.Fe.35 - ~70%). Thus, the thermal denaturation of protein in the studied material could increase their digestibility. This may confirm the thesis that not all proteins contained in the test material are susceptible to the action of digestive enzymes, which may affect their further absorption. Ferritin is a protein resistant to high temperatures, *i.e*., 85° C, low pH and a number of proteolytic enzymes, with confirmed extractivity in pH close to 8.0 [9, 22]. And *in vivo* studies confirm its resistance to digestion [8]. Thus, the presented results suggest possible ferritin extraction among other proteins in the intestine.

### Iron Release

As a next step in the presented experiments, the release of iron during digestion of the studied material was checked. At this stage of experiment L.Fe.35 and S.Fe.35 samples were analyzed, containing 680.4 ± 37.2 mg of total iron/100 g d.m. and 563.4 ± 52.7 mg of total iron/100 g d.m., respectively.

**Fig. 1:**
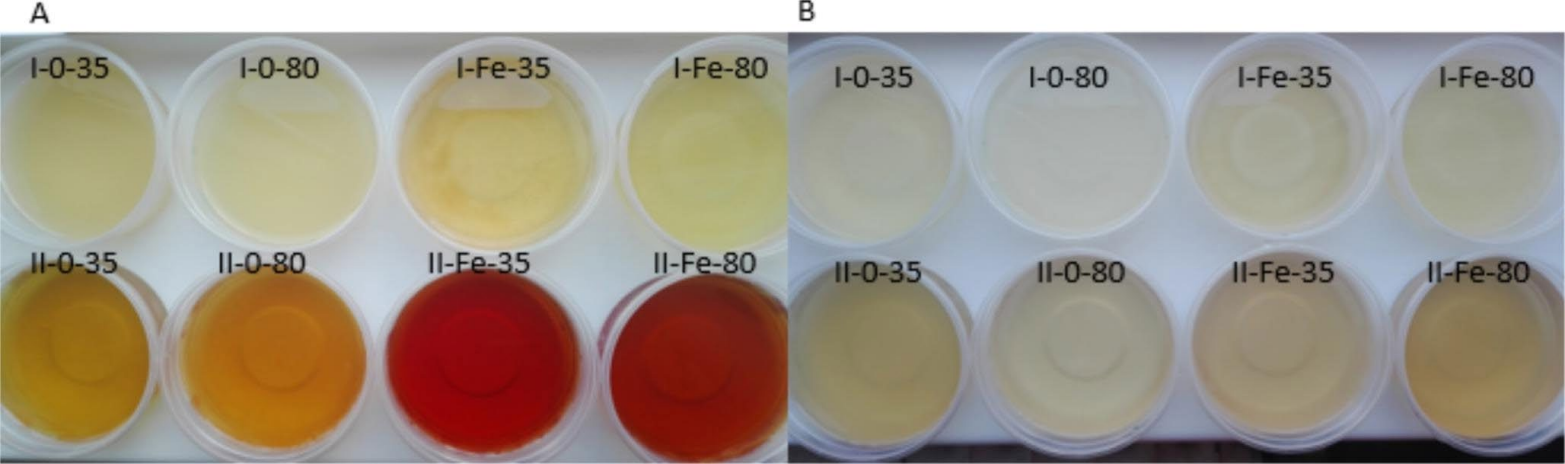
Photography presenting liquids obtained after *in vitro* digestion of A\ lupine B \ soybean sprouts I – liquids taken after the first stage of digestion (stomach) II - liquids taken after the second stage of digestion (after stomach + small intestine digestion); 0 – control sample, Fe – samples enriched in iron; 35^o^C – sprouts dried in the temperature 35^o^C, 80^o^C – sprouts dried in the temperature 80^o^C

Significant differences were observed even in the color of the liquids obtained after digestion of lupine (Fig. 1a) and soybean (Fig. 1b) sprouts. The color observation indicates the release of iron from the tested material in the intestine, and a different form of iron released in the two studied steps of digestion. For the bioavailability of iron from the studied material not only compound resistance to the digestive enzymes may be important, but also their susceptibility to extraction from the food matrix. Following digestion of lupine sprouts, intestine fluid was ‘red’, which suggests presence of ‘red’ iron. That could be the result of the presence both of ferric ions (Fe^3+^) and a complexed form of iron (such as ferritin).

Thus, iron speciation in the obtained digestive fluids was performed. Table 2 shows the content of iron in individual samples.

**Table 2:** The conent of iron [mg] and its form (Fe(II), Fe (III) and complexed iron) released into in the digestive fluid

		mg of iron released during digestion of 1 g sample			
		Total iron	Fe (II)	Fe(III)	Complexed Fe
Lupine sprouts	In the stomach	1.42 ± 0.41	1.22 ± 0.33	ND*	0.21 ± 0.04
	In the intestine	2.52 ± 0.30	0.81 ± 0.15	0.63 ± 0.17	1.08 ± 0.18
Soybean sprouts	In the stomach	0.90 ± 0.17	0.86 ± 0.22	ND	0.04 ± 0.01
	In the intestine	1.30 ± 0.23	0.35 ± 0.14	0.23 ± 0.01	0.71 ± 0.20

*ND- not detected

**Fig. 2:**
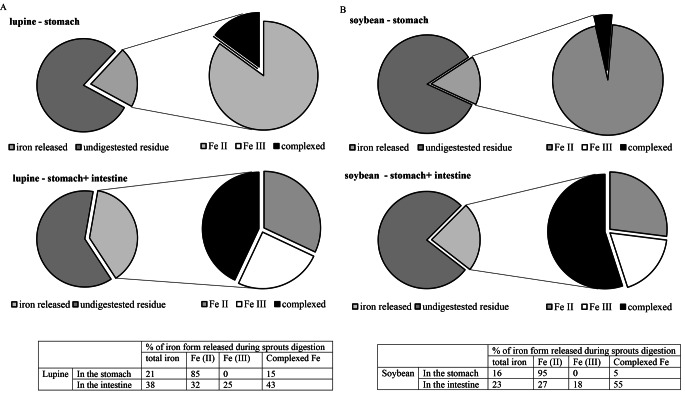
Iron released during digestion of (A) lupine (B) soybean sprouts

During the first step of digestion from the studied material most of released iron was in ionic form, as ferrous iron (85%±3% in lupine and 95%±2% in soybean) (Fig. 2). Moreover, when the same model of digestion was performed without the use of enzymes, solely acid action caused the release of ~ 80% of the iron released during the presented experiment. Intestine digestion allowed to almost double the amount of iron released from lupine sprouts (from ~ 21% up to 38% of total iron), while in soybean it was not such a significant increase (from ~ 16% up to ~ 23%). Ferric iron (which is less available) constituted ~ 25% of total iron in intestine after lupine digestion and ~ 18% after soybean digestion. It suggests that administration of vitamin C together with the prepared sprouts should increase the iron absorption in the intestine. The increased content of complexed iron (Fig. 2) was detected and it constituted ~ 16.5% of total iron for lupine and ~ 12% for soybean sprouts. However, this result still does not prove or exclude the stability of ferritin during digestion. Iron may be complexed here also by polyphenolic compounds, synthetized in time of sprouting; however, it is suggested that in abiotic stress conditions ferritin is the dominating compound binding iron [22–24].

### Isolation of Ferritin from the Digestive Fluids

**Fig. 3:**
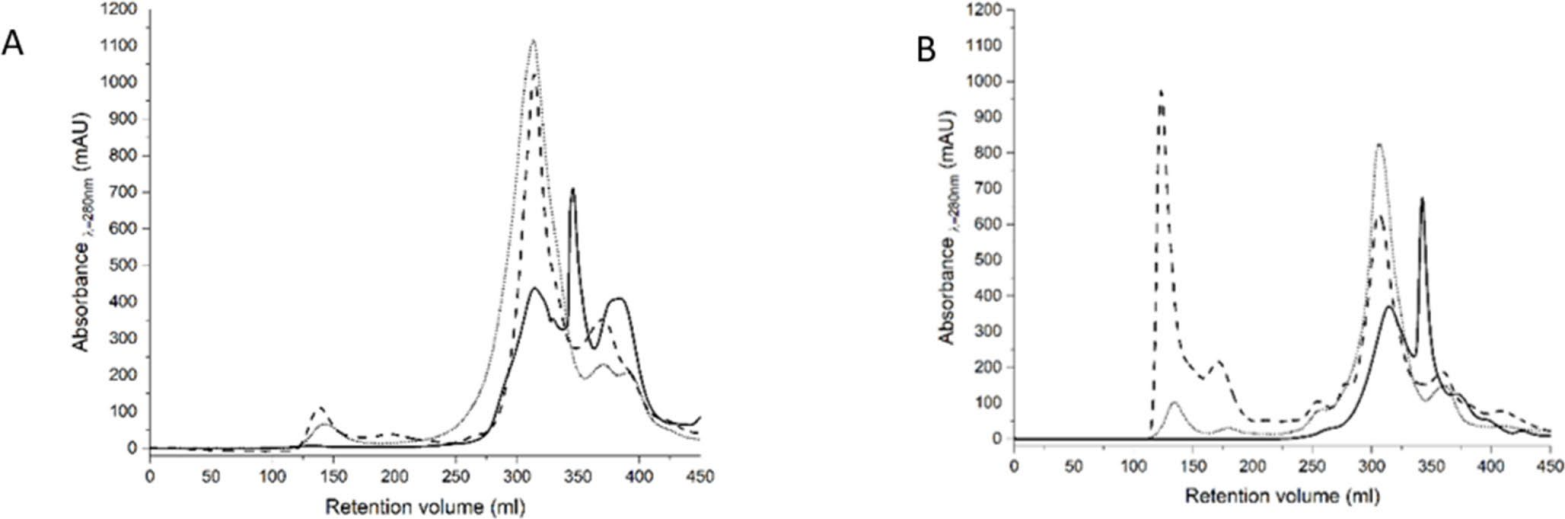
Size exclusion chromatographic analyses of the fluids obtained after lupine (A) and soybean (B) digestion: **―** - stomach (S); **----** - solely intestine (I); **….** – two-step (stomach and intestine) (SI)

Thus, as a next step, chromatographic isolation of ferritin was attempted from the fluids obtained after digestion to confirm or deny the possibility of extraction and stability of ferritin during digestion of the studied samples. Chromatogram presented in Fig. 3A and 3B suggests that ferritin (collected in the fraction between 125 and 155 mL) is not present in the fluid after gastric digestion. This may result mainly from the inability to extract ferritin under such conditions. The best extraction conditions to obtain ferritin fractions with good yield correspond to pH approximately 8.0 [17]. Another essential factor that could have influenced the ferritin level after gastric digestion is the low pH of the environment in which this process takes place. As Bejjani et al [25] demonstrated, pea ferritin dissociates when exposed to a low pH, releasing iron into the digestive fluid simultaneously. Under these conditions, the proportion between the number of *α*-helices and *β*-sheet structures changes, wherein the number of the latter increases significantly. It is worth noting that in the case of the presented results, correction of stomach fluid pH after the end of this step of digestion up to 7.4 did not modify the results. It excludes the possibility of reassembling ferritin degraded in the stomach after modification of pH, as it was observed in other studies [26, 27]. Solely intestine digestion of sprouts, both lupine and soybean, resulted in extraction of ferritin from the material. Moreover, this extraction was many times higher in case of soybean. Simultaneously, after application of two-step digestion of the material, the amount of extracted ferritin decreased, especially for soybean. This suggests the advisability of administering ferritin preparations after encapsulation, limiting its contact with the gastric fluid (*e.g*., in eudragit), which is not consistent with the observations of Theil et al [8]. Intestine condition seems to be more convenient to ferritin extraction, but also safe to maintain its structure and, consequently, to ensure the possibility of its absorption by endocytosis [7]. However, this extraction is still limited – the pH 7.4 is far from pH 8.0 suggested for ferritin extraction.

### The Detection of Ferritin in the Digestive Fluids

**Fig. 4:**
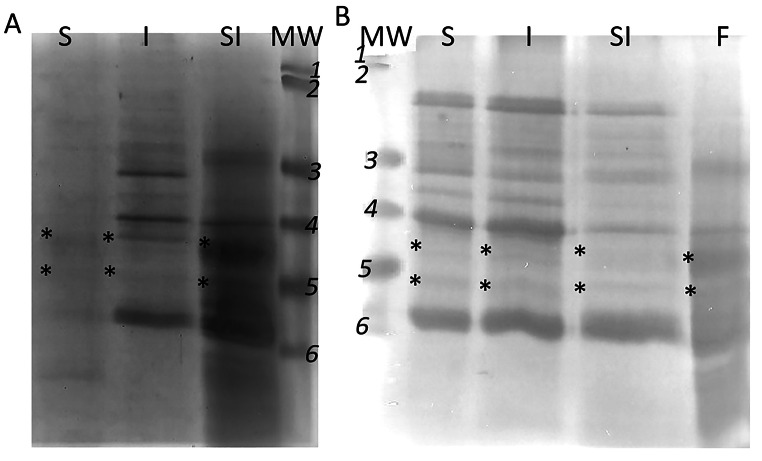
Electrophoregram obtained after separation of fluid after A\ lupine B\soybean S - stomach digestion, I - intestine digestion, SI - two-step digestion (stomach and intestine); F - partially purified lupine ferritin isolate; MW – molecular weight marker (1- 120 kDa, *2*–85 kDa, 3–50 kDa, 4–35 kDa, *5*–25 kDa, − 20 kDa). *- peptide subunits with molecular weight comparable to ferritin subunits

**Fig. 5:**
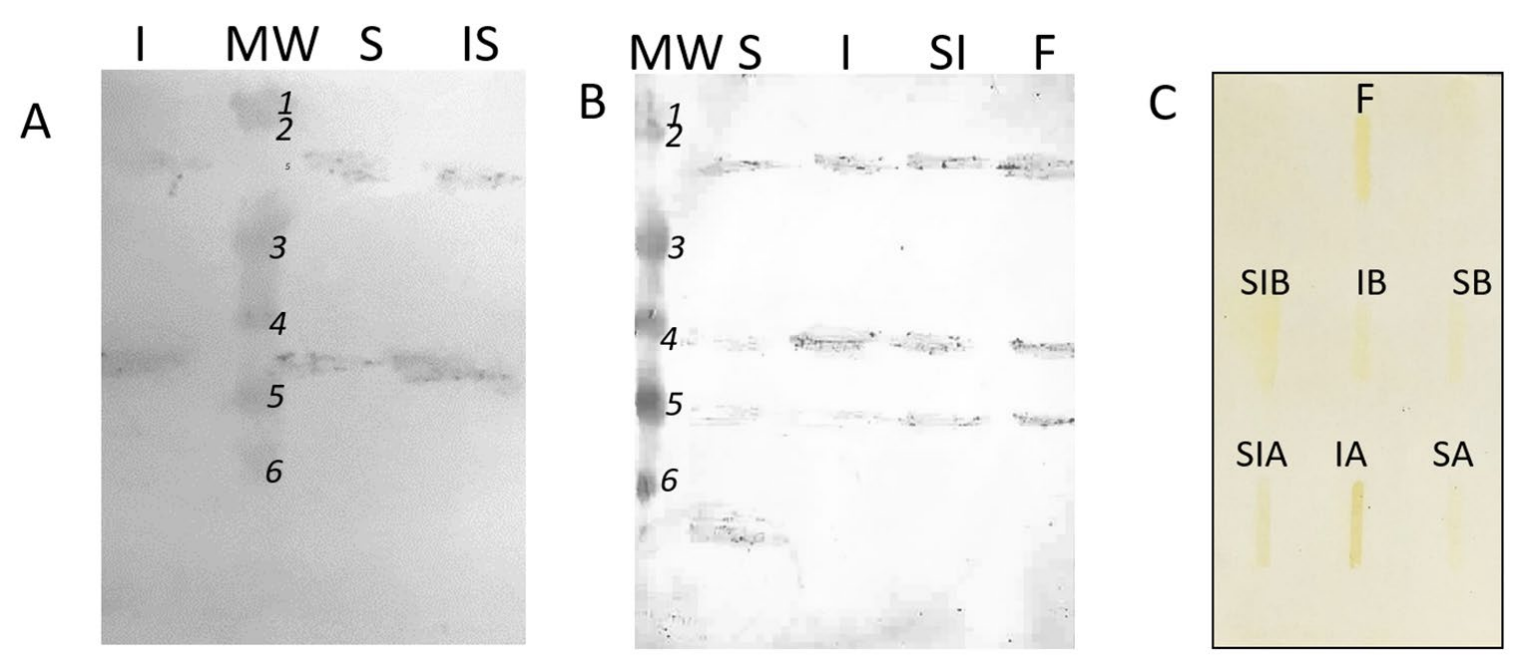
Western blot (A-B) and slot blot analysis (C). Samples of A\ lupine B\soybean after S- stomach digestion, I - intestine digestion, SI - two-step digestion (stomach and intestine); F - partially purified lupine ferritin isolate; MW – molecular weight marker (1- 120 kDa, 2–85 kDa, 3–50 kDa, 4–35 kDa, 5–25 kDa, 6–20 kDa)

The samples were subjected to the SDS-PAGE analysis. Analysis of the obtained gels (Fig. 4) suggest the presence in the studied fraction of peptides with molecular weight close to the molecular weight of ferritin subunits. Molecular weight of ferritin is close to ~ 450 kDa. It is a multimeric protein composed from 16 subunits with molecular weight ~ 28 kDa and 26.5 kDa (the second subunit is the product of deletion of amino acids from the C-end) [22, 28, 29]. However, results of the electrophoretical separation of such not homogenous material (even partially purified ferritin isolate – line F, Fig. 5) could be very misleading, especially in case of such rich in protein material (legume proteins and solution applied to digestion process). Problems in the results of SDS-PAGE interpretation are eliminated by immunodetection, therefore the separated proteins were transferred to the membrane and detected by polyclonal anti-ferritin antibodies. Western blot analysis (Fig. 5A and B) confirmed reaction of the goat serum with the protein present in the fraction separated by FPLC from fluids obtained after digestion of soy sprouts and lupine sprouts. The antibodies recognized even the ferritin subunits in fluids after gastric digestion, which suggests that some ferritin was extracted and denatured during gastric digestion. The same result was observed in slot-blot analysis (Fig. 5C). It must be remembered that samples from gastric digestion had to be concentrated before analysis and the consistency of soy samples from intestine (both solely I, as well as SI digestion) significantly impeded the migration of the sample through the membrane.

## Conclusion

The results presented in this paper confirm our previous observations regarding protein shell dissociation induced by acid in gastric conditions [5, 25]. Simultaneously, these conditions are considered not to be suitable for the extraction of ferritin from complex matrices (plant materials or food). Thus, it may be expected that ferritin did not ‘partially escape’ from stomach digestion [30], but just that it was not extracted (or only partially extracted) in stomach. These explain also differences between the results of *in vitro* studies on pure, isolated ferritin and *in vivo* studies, when ferritin was administered as a food ingredient. Food should be processed to keep ferritin undenatured and easily extracted. This protein is released from the food matrix mainly during digestion at the intestinal stage. Thus, extracted ferritin can be absorbed by endocytosis (by a unique system mechanism in intestinal enterocytes [7]); but in case of denaturation becomes as a source of iron ions transported by DMT1. The results are unique because they do not present studies carried out on isolated ferritin, but on a material containing the protein. They confirmed the protective effect of food ingredients on the stability of ferritin during digestion.

## Electronic Supplementary Material

Below is the link to the electronic supplementary material.

Supplementary Material 1

Supplementary Material 2

Supplementary Material 3

## Data Availability

The datasets generated during and/or analysed during the current study are available from the corresponding author on reasonable request.
