# Supplementary material for: Digestibility of Protein and Iron Availability from Enriched Legume Sprouts
Source: Plant Foods Hum Nutr. 2023 Feb 2;78(2):270–8. doi: 10.1007/s11130-023-01045-x (PMC10363042; doi:10.1007/s11130-023-01045-x)
Supplement: Supplementary file 1 — Supplementary Material 1 [file 11130_2023_1045_MOESM1_ESM.docx]

**Suppl.1** Calibration curve for the FPLC separation

| MW | log MW | Ret. Vol. |  |  | **Protein** | **MW Da** | **Log MW** | **Ret. Vol ml** | **kav** |  | **Vc** |  |
| --- | --- | --- | --- | --- | --- | --- | --- | --- | --- | --- | --- | --- |
| 440 | 2,643453 | 140,31 |  |  | Blue dextran | 2000000 | 6,30103 | **114,82** |  |  | **318** |  |
| 158 | 2,198657 | 169,34 |  |  | Ferrytyna | 440000 | **5,643453** | 140,31 | **0,125455** |  |  |  |
| 75 | 1,875061 | 192,16 |  |  | Aldolasa | 158000 | **5,198657** | 169,34 | **0,268333** |  |  |  |
| 44 | 1,643453 | 209,47 |  |  | Conalbumina | 75000 | **4,875061** | 192,16 | **0,380648** |  |  |  |
| 29 | 1,462398 | 229 |  |  | Ovo | 44000 | **4,643453** | 209,47 | **0,465843** |  |  |  |
| 6,5 | 0,812913 | 270,87 |  |  | Carbonic anhydrase | 29000 | **4,462398** | 228,12 | **0,557634** |  |  |  |
|  |  |  |  |  | Aprotinine | 6500 | **3,812913** | 270,87 | **0,768038** |  |  |  |
|  |  |  |  |  |  |  |  |  |  |  |  |  |
|  |  |  |  |  | **kav = -0.3567 x Log_10_ MW + 2.13** | | |  |  |  |  |  |
|  |  |  |  |  |  |  |  |  |  |  |  |  |
|  |  |  |  |  |  | | | | | | | |
|  |  |  |  |  |  |  |  |  |  |  |  |  |
|  |  |  |  |  |  |  |  |  |  |  |  |  |
|  |  |  |  |  |  |  |  |  |  |  |  |  |
|  |  |  |  |  |  |  |  |  |  |  |  |  |
|  |  |  |  |  |  |  |  |  |  |  |  |  |
|  |  |  |  |  |  |  |  |  |  |  |  |  |
|  |  |  |  |  |  |  |  |  |  |  |  |  |
|  |  |  |  |  |  |  |  |  |  |  |  |  |
|  |  |  |  |  |  |  |  |  |  |  |  |  |
|  |  |  |  |  |  |  |  |  |  |  |  |  |
|  |  |  |  |  |  |  |  |  |  |  |  |  |
|  |  |  |  |  |  |  |  |  |  |  |  |  |
|  |  |  |  |  |  |  |  |  |  |  |  |  |
|  |  |  |  |  |  |  |  |  |  |  |  |  |
|  |  |  |  |  |  |  |  |  |  |  |  |  |
|  |  |  |  |  |  |  |  |  |  |  |  |  |
|  |  |  |  |  |  |  |  |  |  |  |  |  |
|  |  |  |  |  |  |  |  |  |  |  |  |  |
|  |  |  |  |  |  |  |  |  |  |  |  |  |
