# Supplementary material for: Digestibility of Protein and Iron Availability from Enriched Legume Sprouts
Source: Plant Foods Hum Nutr. 2023 Feb 2;78(2):270–8. doi: 10.1007/s11130-023-01045-x (PMC10363042; doi:10.1007/s11130-023-01045-x)

**Fig.4** Electrophoregram obtained after separation of fluid after A\ lupine B\soybean digestion

A)


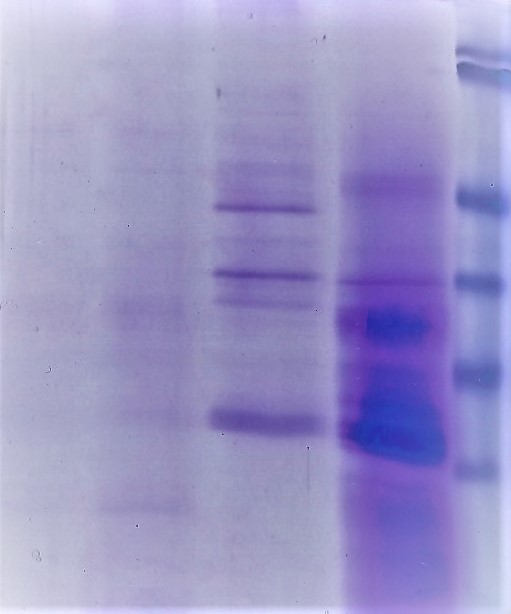


B)


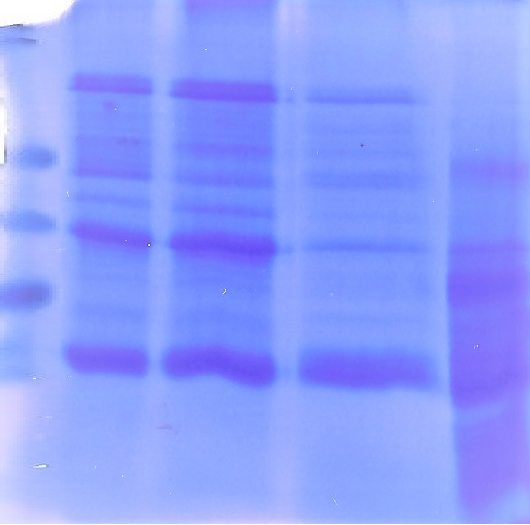

Supplement: Supplementary file 2 — Supplementary Material 2 [file 11130_2023_1045_MOESM2_ESM.docx]
