# Supplementary figures and images for: Digestibility of Protein and Iron Availability from Enriched Legume Sprouts
Source: Plant Foods Hum Nutr. 2023 Feb 2;78(2):270–8. doi: 10.1007/s11130-023-01045-x (PMC10363042; doi:10.1007/s11130-023-01045-x)

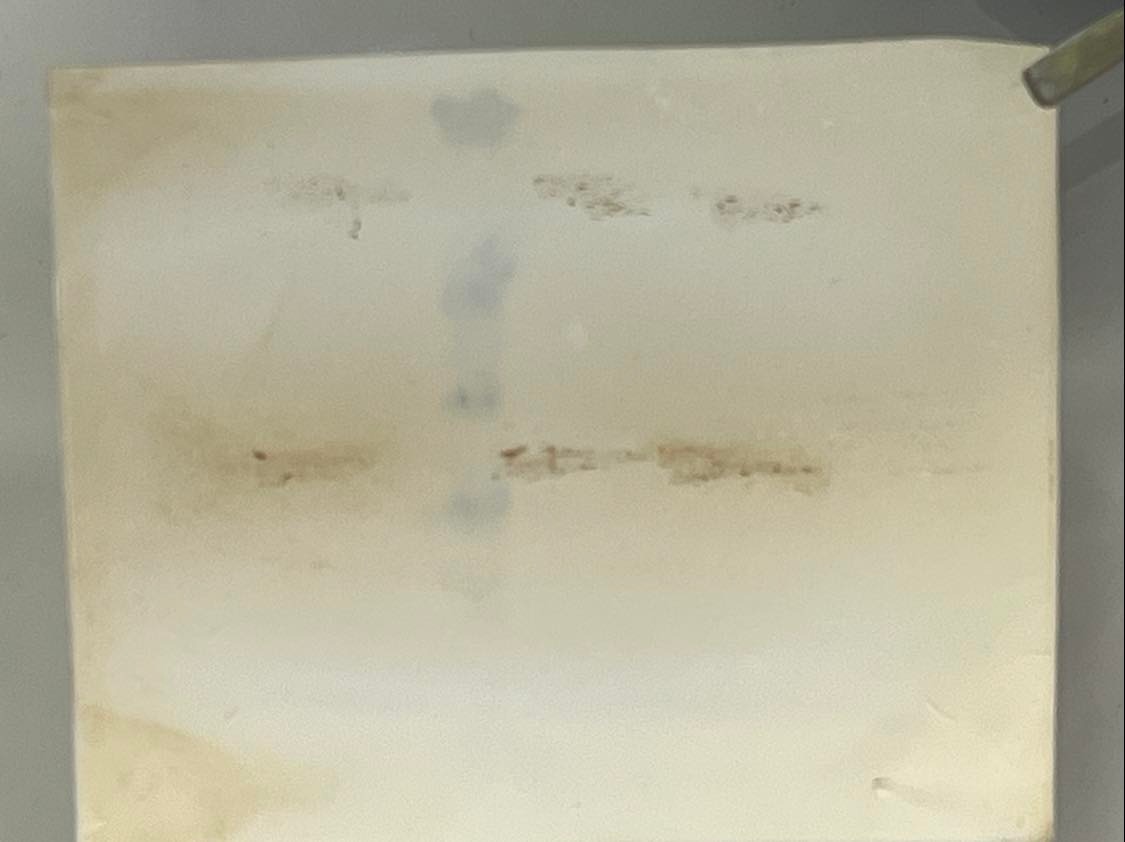


5A)


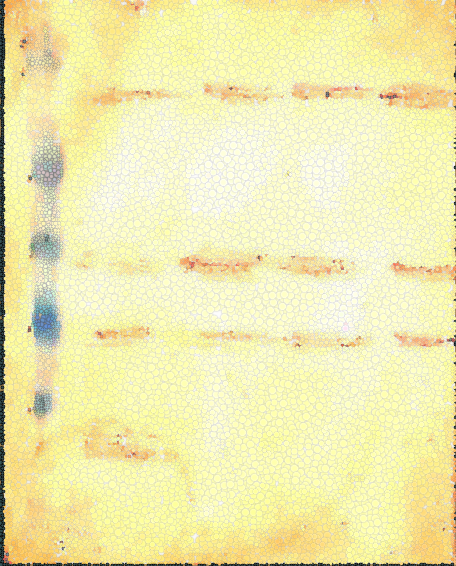


5B)


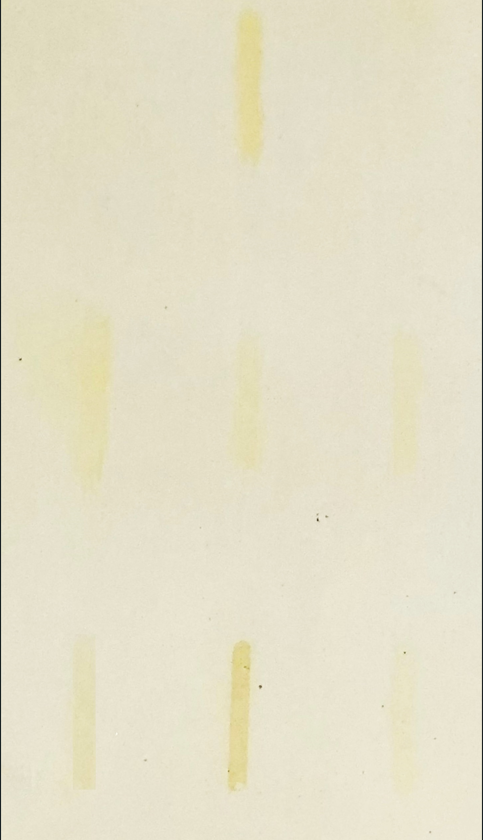


5C)

**Fig.5** Western blot of A\ lupine B\soybean samples and slot blot analysis (C).

Supplement: Supplementary file 3 — Supplementary Material 3 [file 11130_2023_1045_MOESM3_ESM.docx]
